# Obesity and Erectile Dysfunction: Is Androgen Deficiency the Common Link?

**DOI:** 10.1100/tsw.2009.79

**Published:** 2009-07-27

**Authors:** Robert J. Feeley, Abdulmaged M. Traish

**Affiliations:** Department of Biochemistry and Urology, Boston University School of Medicine, Boston, United States

**Keywords:** obesity, dyslipidemia, testosterone, hypogonadism, diabetes, endothelial dysfunction, vascular disease, metabolic syndrome, erectile dysfunction

## Abstract

Obesity is associated with increased risk of erectile dysfunction (ED); however, the underlying causes of ED in obese individuals remain poorly defined. The aim of this review is to discuss the evidence available on the relationship between obesity and ED. A search of published studies in PubMed from 1970 through 2009 was conducted, and relevant articles were evaluated and discussed.

Visceral obesity is a public health threat, and is associated with increased risk of diabetes, vascular disease, endothelial dysfunction, and ED. Plasma testosterone levels are reduced in obesity, further contributing to an increased risk of vascular pathology in obesity. The recognition of the relationship between obesity, reduced testosterone levels, and ED has paved the way for new approaches to manage and treat obese, hypogonadal patients with ED. Obesity profoundly and adversely impacts overall health and, in particular, vascular health, by increasing proinflammatory factors, altering endothelial function and the androgen endocrine milieu, thus increasing the risk of ED.

## INTRODUCTION

Obesity is a major public health concern worldwide. The adverse health consequences of obesity include diabetes, vascular diseases, atherosclerosis, and erectile dysfunction (ED). The prevalence of obesity has increased significantly over the past several decades. Ogden et al.[1] reported that in the U.S., 32.2% of adults were obese. Data from Great Britain, Australia, and Spain also reported similar increases in obesity[2,3,4,5]. Central (abdominal visceral) obesity is characterized by increased waist circumference (WC) and body mass index (BMI). Obesity is a heterogeneous condition; thus, not all obese individuals are expected to exhibit comorbidities associated with obesity.

Erectile function depends on the health of the central and peripheral nervous systems, the vascular bed of erectile tissue, and the hormonal milieu of the individual (reviewed in [6]). Obesity significantly impacts vascular health and alters the balance of the endocrine milieu and contributes to the pathophysiology of ED. Indeed, a link between obesity and ED has been suggested by a number of clinical studies[7,8,9,10,11,12,13]. Obesity-related comorbidities contribute to the impairment of nitric oxide (NO) synthesis in vascular beds, reduce testosterone (T) plasma levels, alter endothelial function, and increase dyslipidemia, all of which contribute to the pathophysiology of ED. In this review, we evaluate the relationship between obesity and ED, and discuss the hypothesis that obesity-related androgen deficiency may be a common link in these two pathophysiological states (Fig. 1).

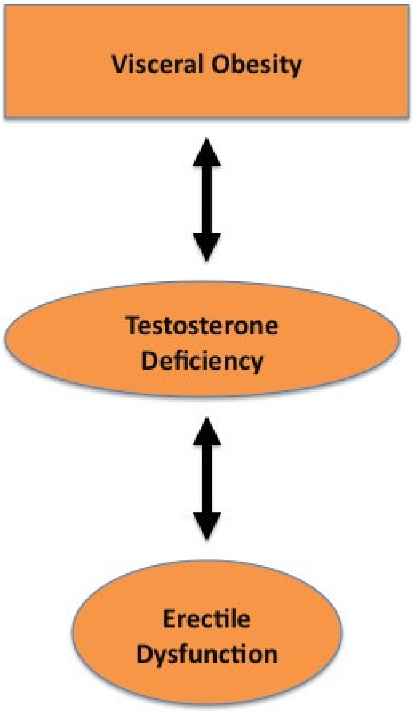

**FIGURE 1.**

## OBESITY AND ED

A host of clinical studies suggest a link between obesity and ED[7,8,9,10,11,12,13]. In the Health Professionals Follow-up Study, maintenance of erectile function was dependent on physical activity and leanness[8]. In addition, men with a BMI < 25 had a 30% lower risk of developing ED than those with a BMI > 28.7. In a subsequent study, Bacon et al.[9] analyzed data from 22,086 men and found that, after 14 years of follow-up, obesity and a sedentary lifestyle significantly contributed to ED. In addition, in a randomized controlled trial, Esposito et al.[10] found that exercise along with a decreasing BMI reversed ED in approximately one-third of obese men. Esposito et al.[14] found that patients with ED tend to be hypertensive and hypercholesterolemic, two conditions often associated with obesity. Blanker et al.[15] investigated the effects of obesity on ED in 1,688 men (ages 50–78). The authors reported that obesity contributed significantly to ED. Cardiac problems, chronic obstructive pulmonary disease, and smoking are independent risk factors for ED. In a community-based cross-sectional study of 1,181 Danish men, Andersen et al.[16] noted that in young (ages 20–45) and old men (ages 50–75), a higher BMI correlated with increasing frequency of ED, with a significant interaction in the younger cohort (odds ratio of 2.74).

Based on the information in the studies cited above, it is clear that obesity is a risk factor for ED. ED is thought to be a warning sign for vascular disease, and a potential marker for atherosclerosis, endothelial dysfunction, and cardiovascular disease (CVD)[17,18]. Insulin resistance and hyperinsulinemia, often found in obese patients, affect sexual function via a decrease in total and free T levels[19,20,21]. Obesity induces alterations in cytokine and adipokine secretions. These proinflammatory factors contribute to endothelial dysfunction and insulin resistance, pathologies that increase the risk of ED[22]. Insulin resistance and obesity are often found together in the same patient, and are components of the metabolic syndrome (MetS). Therefore, it is not surprising that MetS is also associated with increased risk of ED[23,24]). In fact, Esposito et al.[25] found that an increasing number of MetS components correlated with increasing ED severity, suggesting that with an increasing burden on the cardiovascular system, the pathogenesis of ED is more evident. In fact, Mulhall et al.[26] found that vasculogenic ED was a predictor of abnormal stress echocardiography, further supporting the link between ED and vascular disease.

Interestingly, Esposito et al.[27] suggested that endothelial microparticles, or cell-derived submicroscopic membranous vesicles, may be partly responsible for mediating the endothelial dysfunction associated with ED. While this particular study did not focus on ED in obese patients, but rather ED in diabetics, the authors did find that in a multivariate analysis correcting for age, anthropometric indices, glucose, and lipid parameters, endothelial microparticles were the only independent predictor of ED in diabetic men as compared with age-matched nondiabetic potent men. It is of interest to assess levels of endothelial microparticles in obese men and their association with endothelial dysfunction. Diaz-Arjonilla et al.[28] further support a link between obesity and ED when describing results of the Massachusetts Male Aging Study (MMAS), which states that:

> *"The incidence of impotence (mild to severe) occurred in 52% of men aged between 40 and 70 years, and the higher probability of ED after adjusting for age correlated with…the metabolic syndrome. The same cohorts of men were studied after an average of 8.8 years, and the data showed that …BMI significantly predicted the risk of developing ED. Of the 154 patients who were overweight, 41% had moderate or complete ED, which was significantly more than that of leaner patients after controlling for other risk factors…Physical activity was also associated with ED with sedentary men posing the highest risk."*

While many studies have reported a link between obesity and ED, some studies have not observed such a relationship. For example, in the MMAS, there was no association between BMI and ED at baseline[29]. In addition, cross-sectional analysis of 2,126 men from the National Health and Nutrition Examination Survey, after adjustment for cardiovascular risk factors, showed a weakened association between obesity and ED[30].

Many studies have linked obesity with endothelial dysfunction and subsequent ED. Endothelial dysfunction negatively influences endothelial-derived NO, which is critical for endothelium-dependent vasodilation[9]. The presence of ED warrants screening for vascular disease, as well as for blood pressure, lipids, and glucose. Corona et al.[31] found that hypertension, in particular, is one of the most important determinants of arteriogenic obesity-associated ED. It has also been shown that weight loss improves endothelial function in obese subjects and subsequent ED. Cho et al.[32] studied the relationship between body fat mass (BFM), measured via bioelectrical impedance, and ED in 208 Korean men (aged 67.4 ± 8.2). The authors used BFM, and not BMI, as a measure of obesity in the geriatric population *"because decrease of height and loss of muscle mass accompany geriatric obesity."* The authors found a U-shaped relationship, such that obese patients, as well as patients with a low BFM, may be at increased risk for ED in an Asian population.

While obesity represents a risk factor for ED, exercise and obesity interventions have been shown to have a beneficial impact on ED[33,34,35]. In addition, therapeutic interventions might also focus on increasing circulating adiponectin levels. Sowers[36] suggested that circulating adiponectin levels are inversely related to tumor necrosis factor-alpha (TNFα) and C-reactive protein (CRP), that adiponectin reduces endothelial cell apoptosis and expression of adhesion molecules, and that weight loss in obese individuals leads to an increase in its expression.

## OBESITY AND ANDROGEN DEFICIENCY

A considerable number of studies show that obesity is associated with androgen deficiency[37,38, 39,40,41,42,43,44,45,46,47,48,49,50,51]. In addition, hypogonadism is directly associated with MetS[45,46,52]. It is unclear whether androgen deficiency precedes obesity or vice versa. Some studies suggest that obesity leads to androgen deficiency. For example, Derby et al.[47] found that total and free T levels declined in men who were consistently obese during 8–9 years of follow-up in the MMAS. Additionally, weight loss has been found to increase circulating T levels[53,54]. Chen et al.[55] studied androgen status and the MetS in men ≥ 70 years old, and suggested that androgen deficiency is a consequence of, and not a cause of, abnormal metabolic status. The exact mechanism that accounts for this link between obesity and androgen deficiency may involve the action of insulin, leptin, or aromatase. Insulin stimulates T production; thus, insulin resistance in obese men may account for a decrease in T production[56]. Testicular tissue expresses leptin receptors and binding of leptin may reduce androgen biosynthesis, which may be a mechanism for the observed hypogonadism in obese men[57]. Loves et al.[58] showed that letrozole, an aromatase inhibitor, increases T and LH levels in severely obese hypogonadal men. Therefore, it seems likely that increased aromatase activity in obesity may be the mechanism by which T levels are reduced since it is metabolized into estradiol. The proinflammatory state that is observed in obese patients may also contribute to T decline. Furthermore, increased inflammatory factors in response to obesity and diabetes alter T biosynthesis. For example, Morales et al.[59] showed that intratesticular TNFα delivery decreased hCG-stimulated T production. Increased estrogen production in obese men has also been shown to influence the gonadotropin releasing hormone (GnRH) LH response and reduces T synthesis[60].

Obesity is associated with low serum T levels[61] as well as reduced SHBG (sex hormone-binding globuline) levels[50,51]. Since it is generally accepted that there is a direct relationship between total T and SHBG, it is reasonable to suggest that the obesity-associated reduction in T levels is due to both a reduction in T biosynthesis as well as in SHBG levels. Such an interaction may result in reduced free T or bioavailable T. It has also been suggested that BMI is negatively correlated with free T and bioavailable T[62,63,64]. Corona et al.[31] showed that even after adjusting for obesity-related factors, obese men with ED had a reduction in SHBG-bound and -unbound T, suggesting that androgen deficiency can result from obesity in and of itself. Interestingly, Tsai et al.[65] reported that both subcutaneous and total body fat, in addition to visceral fat, are associated with decreased total and free T. Nielsen et al.[66], however, showed that this inverse relationship for subcutaneous and total body fat, as opposed to visceral fat, was accounted for by low SHBG levels. It should be noted that not all studies have measured serum T levels in a consistent manner.

## OBESITY AND ENDOTHELIAL DYSFUNCTION

Obesity has been shown to be an independent risk factor of CVD, despite the known associations with other cofactors of blood pressure, blood sugar, and lipids[67,68,69]. However, it is less clear whether it is the obesity *per se* or these associated cofactors that lead to an increase in mortality. Barter et al.[70] found that not all overweight patients display a uniform dyslipidemic profile. These authors identified two groups of overweight individuals, with comparable BMIs, and stratified them according to whether they had high triglyceride and low HDL-c values. The dyslipidemic group, as compared to the control group, had a significantly greater WC, higher levels of CRP, lower levels of adiponectin, as well as higher fasting plasma glucose, insulin, and homeostasis model of assessment-insulin resistance values. It is thought that an increase in WC may contribute to this dyslipidemia via excess visceral fat, which is metabolically different than fat residing in a subcutaneous region. However, even after controlling for WC, Barter et al.[70] observed the multiple metabolic abnormalities in dyslipidemic individuals. In the context of these results, this suggests that WC is an imprecise measure of metabolic dysregulation. It is also possible that the dyslipidemic group may have a genetic predisposition for insulin resistance that extends beyond adipose tissue to include muscle and the liver.

Diaz-Arjonilla et al.[28] suggested that endothelial dysfunction is triggered by a cascade of inflammatory responses arising from adipose tissue activation in obese individuals. Specifically, inflammatory-mediated factors, such as leptin, resistin, IL1, IL6, TNFα, and adiponectin, are implicated in endothelial dysfunction and subsequent CVD. Adiponectin is an anti-inflammatory agent and is known to protect against atherosclerosis, and it has been observed that adiponectin levels are reduced in obese individuals[71]. In contrast to adiponectin, leptin levels are elevated in obese individuals and it has been shown that high leptin levels contribute towards endothelial dysfunction as well as atherosclerosis[22,72]). Guzik et al.[22] also suggested that obesity-induced elevated resistin levels promote increased macrophage infiltration into tissues via an up-regulation of adhesion molecules and increased cytokine production.

Giugliano et al.[73] investigated whether an association existed between erectile function, endothelial function, and markers of systemic vascular inflammation in 80 obese men (ages 35–55). The patients were divided into two subgroups according to ED diagnosis. The authors reported that, in comparison to nonobese controls, all obese men had impaired endothelial function as well as elevated IL6, IL8, IL18, and CRP. They also observed that obese men with ED displayed greater endothelial dysfunction in comparison to obese men without ED. The authors suggested that a common pathway involving impaired NO activity may underlie the observed endothelial dysfunction and associated ED.

It is known that obesity and MetS are characterized by low-grade chronic inflammation and oxidative stress. In fact, macrophage infiltration into intra-abdominal fat, as opposed to subcutaneous fat, has been shown to correlate with increased monocyte chemoattractant protein-1 (MCP1) and colony stimulating factor-1 (CSF1) mRNA levels, which may be partly responsible for linking central adiposity to increased risk for CVD[74]. Free radical formation may influence the integrity of the endothelium by reducing NO bioavailability[75], and Roberts et al.[76] reported that obese men who diet and exercise are capable of increasing NO bioavailability. CRP levels have also been found to be inversely related to NO bioavailability and to have direct effects on the vessel wall, which contributes to atherosclerosis[77]. Increased erythrocyte aggregation, which increases blood viscosity and affects the microcirculation, has also been put forth as a link between obesity, ED, and coronary artery disease[78].

Obesity has a profound effect on the endocrine milieu, and the resulting proinflammatory responses lead to insulin resistance and endothelial dysfunction. Evidence exists that suggests that low T is an independent predictor of endothelial dysfunction in men[79]. Therefore, androgen treatment may also help to reduce endothelial dysfunction, especially as hypogonadal men have a reduced number of circulating endothelial progenitor cells (EPCs)[80] and androgen treatment has been shown to stimulate EPCs through an androgen receptor-mediated pathway[81].

## SUMMARY AND CONCLUSIONS

Data from a host of clinical studies have established that an association exists between visceral obesity, androgen deficiency, endothelial dysfunction, and ED; however, the causal relationship between these variables remains unknown.

Considerable evidence links obesity with reduced T levels, resulting in a hypogondal state, which is a risk factor for ED. Nevertheless, the pathophysiological mechanisms that underlie these conditions require additional investigation. Here, we advance the hypothesis that visceral obesity contributes to androgen deficiency, thus increasing the risk of ED.

## ACKNOWLEDGMENT

This work was supported by the Departments of Biochemistry and Urology, Boston University School of Medicine
